# 3D-Printed Mucoadhesive Hydrogel Buccal Films Based on HPMC and Carbopol Bioinks Incorporating Cyclodextrin–Cannabinoid Complexes and Terpenes

**DOI:** 10.3390/gels12050386

**Published:** 2026-05-01

**Authors:** Anushree Nagaraj, Ali Seyfoddin

**Affiliations:** School of Science, Faculty of Health and Environmental Sciences, Auckland University of Technology, Auckland 1010, New Zealand

**Keywords:** 3D printing, hydrogel bioinks, mucoadhesive gels, cannabinoid delivery, buccal drug delivery, cyclodextrin inclusion complexes, terpenes, permeation enhancers, semi-solid extrusion printing, polymeric gel matrices

## Abstract

Three-dimensional (3D) printing has emerged as a versatile platform in pharmaceutical sciences, enabling fabrication of personalized dosage forms with controlled drug release and tailored properties using printable hydrogel bioinks. This study aimed to develop mucoadhesive hydrogel buccal films for cannabinoid delivery using extrusion-based 3D bioprinting. The films incorporated cannabidiol (CBD) and tetrahydrocannabinol (THC) as cyclodextrin inclusion complexes with HPMC or Carbopol as mucoadhesive hydrogel-forming polymers, while terpenes were evaluated as permeation enhancers. Terpenes including 1,8-cineole, d-limonene, α-pinene, and L-menthol were investigated individually and in combinations to assess their ability to enhance buccal cannabinoid permeation. Hydrogel bioinks were prepared and characterized for viscosity, pH, and drug content prior to printing under optimized conditions. The printed films were evaluated for mechanical properties, swelling behaviour, mucoadhesion, in vitro drug release, and ex vivo buccal mucosal penetration. Ex vivo penetration studies demonstrated that combinations of natural terpenes significantly improved CBD penetration compared with individual terpenes and the synthetic enhancer Azone. HPMC-based hydrogel films exhibited superior mechanical strength, cohesive gel matrices, and sustained non-Fickian cannabinoid release, while enhancing transmucosal penetration compared with unformulated drugs. Carbopol-based films showed higher mucoadhesion but weaker mechanical properties and faster erosion-driven release. These findings demonstrate the potential of 3D-printed mucoadhesive hydrogel films as gel-based systems for transmucosal cannabinoid delivery.

## 1. Introduction

Cannabinoids such as cannabidiol (CBD) and tetrahydrocannabinol (THC) possess therapeutic potential in curbing diseases and alleviating symptoms of various pathologies [1]. An increasing number of countries are approving the administration of regulated cannabinoid products for various conditions, including spasticity associated with multiple sclerosis (MS), rare epileptic disorders Lennox–Gastaud and Dravet syndromes, chronic pain, chemotherapy-induced emesis and nausea, and reviving appetite in patients affected by human immunodeficiency virus (HIV) [2]. However, their clinical effectiveness is limited by biopharmaceutical constraints. Classified as Biopharmaceutics Classification System (BCS) Class II drugs, cannabinoids exhibit extremely low aqueous solubility and high lipophilicity, resulting in dissolution-limited absorption, especially in non-intestinal environments such as the buccal cavity [3,4]. Buccal administration offers direct entry into systemic circulation while bypassing first-pass metabolism, a larger surface area through which significant volume of drugs can be delivered, and high patient compliance [5,6], but the physicochemical properties of cannabinoids restrict mucosal permeation and bioavailability [7,8]. Therefore, innovative formulation strategies that concurrently enhance solubility and improve mucosal permeation are essential for efficient buccal delivery.

To overcome the solubility barrier of cannabinoids, cyclodextrin-based complexation, particularly with hydroxypropyl-β-cyclodextrin (HP-β-CD), is well established in the literature as an efficient approach to increase cannabinoid dissolution, stability, and membrane interaction. Previous studies have demonstrated several-hundred-fold increases in solubility using HP-β-CD complexes and rapid dissolution under aqueous conditions, supporting their use as a dispersion-enhancing carrier system for transmucosal delivery [9,10,11,12]. Beyond solubility enhancement, effective buccal transport of cannabinoids requires modulation of the mucosal barrier. Terpenes are effective permeation enhancers, known for their ability to increase the absorption of various compounds across biological membranes. They achieve this by interacting with the lipid layers of cell membranes, disrupting their structure, and enhancing the fluidity of the membrane. This disruption creates temporary openings or makes the membrane more permeable, thereby facilitating drug permeation [13]. They are naturally occurring, less toxic than chemical enhancers and have the potential to produce effects with minimal and reversible irritation to the tissue when used in low concentrations of 1% to 5%. They are also recognized as generally safe by the US FDA [13,14,15]. Terpenes, such as limonene, 1,8-cineole, menthol, and α-pinene have been recognized for their permeation enhancing effects of lipophilic drugs in oro-mucosal and transdermal drug delivery systems [16,17,18,19]. These four terpenes were selected based on their varying lipophilicity and boiling points, enabling investigation of their differential interactions with mucosal lipid domains and their contribution to the pull–push permeation mechanism. Using a combination of terpenes, rather than relying on a single terpene, could have the potential to increase the absorption rate of drugs through the buccal mucosa. Numerous studies have established combinations of terpenes in various concentrations to be effective in transdermal permeation of lipophilic drugs [20]. The ability of combined terpenes to enhance the permeation of lipophilic drugs through the buccal mucosa remains limited, necessitating further research to better understand their potential.

Three-dimensional (3D) printing has emerged as a transformative technology in pharmaceutical sciences, facilitating the rapid fabrication of complex, personalized therapeutics with high precision. This approach is particularly advantageous for a diverse range of drugs, as it allows meticulous control over drug loading, uniform distribution, and release kinetics, ensuring consistent and predictable therapeutic outcomes. Also referred to as additive manufacturing, 3D printing constructs dosage forms through a layer-by-layer deposition process, enabling intricate geometries and tailored drug delivery profiles. Compared to conventional solvent casting, extrusion-based 3D printing offers superior control over spatial drug distribution, layer thickness, and internal microstructure. This enables precise modulation of drug release kinetics and mechanical properties, which is particularly advantageous for hydrophobic drugs such as cannabinoids that require uniform dispersion within a polymer matrix. The practical applicability of 3D printing for producing personalized pharmaceutical dosage forms has been formally recognized, as demonstrated by the FDA’s approval of Spritam (levetiracetam) in 2015. This 3D-printed orodispersible tablet highlighted the ability of additive manufacturing to create complex, patient-friendly dosage forms with precise drug content, rapid disintegration, and tailored release characteristics, opening opportunities in 3D-printed pharmaceuticals [21,22,23,24].

A variety of 3D printing technologies have been employed in pharmaceutical sciences to develop innovative dosage forms. Among the most established methods, Fused deposition modelling (FDM) extrudes thermoplastic drug–polymer filaments layer by layer but is limited by high temperatures, making it unsuitable for thermolabile drugs [25,26]. Semi-solid extrusion (SSE) overcomes this by printing drug-loaded hydrogel or gel-based bioinks at ambient or slightly elevated temperatures, allowing incorporation of sensitive actives and widely used for oral films, chewables, and personalized pediatric medicines [27,28]. Laser-based methods such as selective laser sintering (SLS) and stereolithography (SLA) offer high resolution and tunable porosity but require heat-stable drugs and compatible photopolymers [29,30]. Binder jetting and direct powder extrusion (DPE) provide lower thermal input, though challenges remain in achieving uniform drug distribution [31]. These technologies enable dosage forms from fast-dissolving tablets to controlled-release systems, highlighting the versatility of 3D printing in personalized medicine. While these technologies demonstrate the feasibility of additive manufacturing for pharmaceuticals, their reliance on thermoplastics, resins, or powders presents challenges in incorporating mucoadhesive polymers and hydrophilic drug carriers. To address this, extrusion-based 3D bioprinting has emerged as a promising alternative (alternative [32,33,34]. Bioprinting (extrusion-based) is an advanced form of 3D printing that is widely used for the fabrication of biomaterials and scaffolds and has also shown potential for developing drug delivery systems under mild conditions such as the absence of heat, making it suitable for sensitive active pharmaceutical ingredients (APIs). This technique relies on bioinks, which are printable materials such as hydrogels or polymer solutions that can be extruded under mild conditions to form films, patches, or scaffolds suitable for drug delivery [35].

Within mucosal drug delivery, particularly via the buccal route, extrusion bioprinting offers unique advantages by enabling the fabrication of mucoadhesive films, patches, or scaffolds with precise control over thickness, porosity, and drug loading. Buccal films are advantageous because they are thin, flexible, and discreet, which improves patient compliance across vulnerable populations such as pediatric, geriatric, and dysphagic patients. Their ease of administration, without the need for water, further enhances convenience and acceptability. Importantly, bioprinting allows the tailoring of these films to achieve either rapid drug release for immediate therapeutic action or sustained release for prolonged exposure, depending on the therapeutic requirements [36]. Several studies have demonstrated the potential of extrusion-based 3D printing and bioprinting for single or multi-layered buccal film fabrication, highlighting their utility in precise drug deposition and individualized therapy [25,37,38].

The incorporation of mucoadhesive polymers in buccal film formulations significantly enhances their performance by ensuring intimate contact with the buccal mucosa, thereby prolonging residence time and enabling controlled drug delivery. These polymers facilitate the adhesion of the film to the mucosal surface, allowing for sustained drug release and improved therapeutic efficacy. Various mucoadhesive polymers have been utilized as the backbone of buccal films, including natural, synthetic, and semi-synthetic polymers. Natural polymers such as chitosan and sodium alginate offer biocompatibility and biodegradability, while synthetic polymers like polyacrylic acid (Carbopol) and polyvinyl alcohol provide desired mechanical properties and mucoadhesive strength. Semi-synthetic polymers, such as hydroxypropyl methylcellulose (HPMC), combine the advantages of both natural and synthetic polymers, offering versatility in formulation design. Such polymers in addition with other excipients used can be used achieve optimal film characteristics tailored to specific drug delivery requirements [25,39,40].

Additive manufacturing techniques have substantially advanced cannabinoid formulation strategies by enabling precise control over drug dosage, release kinetics, and structural design in pharmaceutical applications. Several studies have demonstrated the potential of 3D printing for cannabinoid delivery. Gościniak, Kocaj [41] fabricated bi-gel systems of cannabidiol (CBD)-rich hemp extracts with hyaluronic acid, while Antezana, Municoy [42] developed gelatine-alginate scaffolds loaded with Cannabis sativa oil for enhanced wound repair, and Jennotte, Koch [43] used FDM to fabricate immediate-release oral CBD dosage forms with tailored solid dispersions. To address the poor solubility and limited bioavailability of cannabinoids, various strategies such as complexation with cyclodextrins or entrapment in nanoparticles have been explored. Andriotis, Monou [44] developed pectin-honey inks containing CBD–β-cyclodextrin complexes, which were printed using semi-solid extrusion-based printing for oral drug delivery. Similarly, Monou, Mamaligka [45] fabricated alginate films incorporating CBD/CBG nanoparticles to support wound healing due to their bioactive and mucoadhesive properties. Despite these advances, research on 3D-printed films for buccal cannabinoid delivery remains limited. Research by Abdella, Kim [22] addressed this by producing hydroxyethyl cellulose (HEC)-based gels loaded with CBD nanostructured lipid carriers (NLCs) using pressure-assisted micro-syringe extrusion, yielding thin, mucoadhesive films suitable for buccal administration.

Building on these previous findings, in this study, various combinations and concentrations of terpenes were evaluated to enhance the ex vivo permeation of cannabinoids using porcine buccal mucosa, and extrusion-based bioprinting was employed to fabricate mucoadhesive buccal films of cannabinoids. Cannabinoids were incorporated as cyclodextrin inclusion complexes to enhance solubility and stability, while HPMC or Carbopol served as mucoadhesive hydrogel-forming polymers to prolong residence time on the buccal mucosa, and optimized terpene combinations. This approach aims to combine the precision and versatility of 3D bioprinting with mucoadhesive hydrogel matrices to achieve controlled release, sustained mucoadhesion, and improved bioavailability of cannabinoids via the buccal route, while also promoting high patient compliance.

## 2. Results and Discussion

### 2.1. Ex Vivo Penetration of CBD in Presence of Terpenes

**Figure 1:**
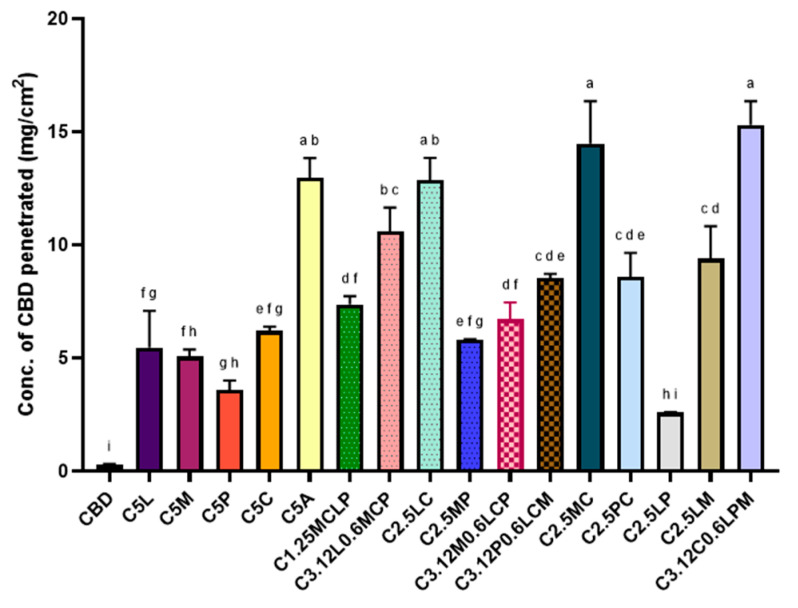
Ex vivo buccal penetration of CBD from formulations listed in Table 1. Data represent mean ± SD (*n* = 3). Different letters indicate statistically significant differences (*p* < 0.05, one-way ANOVA with Tukey post hoc test).

Previous experimental investigations demonstrated that the quantity of CBD that successfully permeated through the mucosal tissue into the receptor compartment was minimal or negligible under ex vivo conditions. Additionally, analysis of the donor compartment revealed that the residual concentration of CBD was also low, indicating that the compound was not simply retained in the donor media. Given these findings, it was hypothesized that CBD may be predominantly localizing within the mucosal tissue itself rather than undergoing full transmembrane diffusion. Hence, CBD penetration was evaluated by quantifying the extent of CBD that partitioned into and diffused throughout the mucosal layers. Ex vivo penetration of CBD in the presence of different concentrations and combinations of terpenes d-limonene, l-menthol, α-Pinene, and 1,8-cineole were compared with chemical permeation enhancer Azone and pure CBD dissolved in MCT vehicle (carrier), as shown in Figure 1. Among all the tested terpenes at a 5% concentration, 1,8-cineole demonstrated the most significant enhancement of CBD penetration into the mucosal tissue. This was followed, in decreasing order of effectiveness, by d-limonene, l-menthol, and α-pinene. None of the terpenes exhibited any inhibitory effect on CBD penetration.

Previous studies have suggested that the lipophilicity and boiling point (BP) of terpenes are critical physicochemical parameters influencing their ability to act as permeation enhancers. Generally, lipophilic terpenes are more effective in facilitating the permeation of hydrophobic drugs like CBD due to their effectiveness in penetrating lipid-rich layers of the skin or mucosa [46]. Longgos, Pequiro [47] investigated the permeation-enhancing effects of various terpenes on lipophilic drugs and found that limonene exhibited greater enhancement compared to menthol, primarily due to its higher lipophilicity. Another key factor influencing drug permeation was the boiling point of the terpenes. Terpenes with higher boiling points tended to show reduced enhancement potential, likely because their increased cohesiveness limited their interaction with biological membranes [48]. Although 1,8-cineole has low lipophilicity, its boiling point is the lowest which might have contributed to higher permeation of CBD. This behaviour can be explained using a ‘pull–push’ mechanism, where lipophilic terpenes enhance drug partitioning into the mucosa (‘pull’), while lower boiling point terpenes increase membrane fluidity (‘push’), facilitating deeper penetration. A previous study by Tabboon, Pongjanyakul [49] reported either negligible or inhibitory effects on mucosal CBD permeation when highly lipophilic terpenes such as nerolidol and oleic acid were used in combination with lipophilic carriers like MCT oil. This was attributed to CBD’s strong affinity for the vehicle, which hindered its partitioning into the buccal mucosa. Table 1. summarizes the log *p* values (indicating lipophilicity) and boiling points of the studied terpenes. While the synthetic enhancer Azone showed greater enhancement effects compared to individual terpenes, almost all combinations of terpenes outperformed Azone significantly. The most effective formulation consisted of 3.125% 1,8-cineole, 0.625% d-limonene, 0.625% L-menthol, and 0.625% α-pinene, which yielded the highest CBD permeation enhancement.

**Table 1:** Log P and boiling point values of terpenes used in this study.

Terpene	Log P	Boiling Point	Reference
1,8-Cineole	2.82	174	[50]
L-Menthol	3.20	215.4	[50]
α-Pinene	2.11	188.6	[50]
D-limonene	4.45	175.4	[51]

### 2.2. Viscosity of Prepared Bioinks

**Figure 2:**
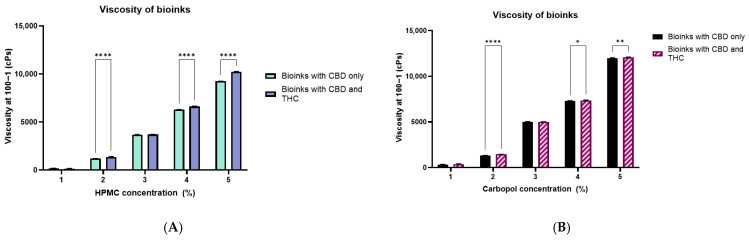
Apparent viscosity profiles of bioinks containing either CBD-only or combined CBD and THC inclusion complexes at 100 s^−1^; (**A**) HPMC-based and (**B**) Carbopol-based bioinks. Data are presented as mean ± SD (*n* = 3). Statistical significance between CBD-only and CBD+THC formulations is indicated (* *p* < 0.05, ** *p* < 0.01, **** *p* < 0.0001).

The viscosity of HPMC- and Carbopol-based bioinks (1–5% *w*/*v*) was evaluated at 100 s^−1^ shear rate to determine the influence of polymer concentration suitable for bioink preparation. In both polymer systems, viscosity increased in a concentration-dependent manner, shown in Figure 2. Carbopol-based bioinks exhibited greater viscosity than HPMC-based bioinks, consistent with prior studies indicating that Carbopol is more viscous than HPMC at equivalent concentrations [52,53].

In both the bioinks, the addition of THC alongside CBD consistently increased viscosity compared to CBD-only formulations, indicating increased interactions from the added THC-cyclodextrin complex strengthening the polymer network in solution. These findings confirm that both polymer concentration and drug loading significantly influence rheological behaviour. From a formulation perspective, viscosity must be carefully optimized, as excessively low viscosity results in poor print fidelity and inadequate mucoadhesion, while overly high viscosity impairs extrusion, increases nozzle clogging risk, and may reduce patient acceptability or drug release rates [54,55]. Based on these considerations, 3% and 4% concentrations of both the polymers were selected for printing buccal films. Selected polymer concentration and drug composition is presented in Table 2.

### 2.3. Three-Dimensional Printing

#### Concentration of Plasticizer (PEG) on Printability of Buccal Films

**Figure 3:**
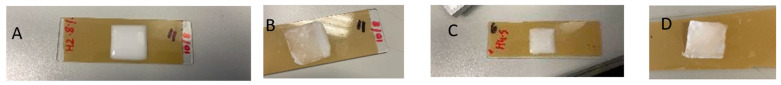
Effect of PEG concentration on the morphology of 3D-printed films before and after drying. (**A**) Film printed with 10% PEG before drying; (**B**) film printed with 10% PEG after drying; (**C**) film printed with 5% PEG before drying; (**D**) film printed with 5% PEG after drying.

The influence of PEG concentration on the printability and film quality of HPMC-based bioinks was investigated as shown in Table 3. Previous research has indicated that PEG levels are crucial to balance extrusion performance with final film stability [56]. At 10% PEG, HPMC-based inks printed well (Figure 3A), but produced sticky films upon drying as shown in Figure 3B, suggesting excess plasticizer compromised structural stability. In contrast, as highlighted in Figure 3C,D, reducing the PEG concentration to 5% resulted in smooth, uniform prints with stable films after drying. Based on these findings, 5% PEG was selected as the optimal plasticizer concentration for further studies. This concentration was subsequently employed in both HPMC- and Carbopol-based bioinks to balance print fidelity, mechanical strength, and post-drying stability of the 3D-printed buccal films. It should be noted that the final bioink composition predominantly consists of PEG combined with relatively low concentrations (3–4% *w*/*v*) of linear polymers (HPMC or Carbopol). Therefore, the rheological and mechanical behaviour of the system is governed by polymer–plasticizer interactions rather than polymer concentration alone, which should be considered when interpreting printability and film stability.

The printing parameters, including pressure and speed, were optimized to achieve continuous filament extrusion of the material/bioink, along with temperature and nozzle specifications used during printing are summarized in Table 4.

All inks were printed at ambient conditions (around 20–24 °C), with only minor variation across formulations. The narrow range indicates that temperature was well controlled and unlikely to have significantly influenced printability differences. A constant nozzle size (25G) and print speed (6 mm·s^−1^) were used for all formulations, meaning that differences in extrusion pressure directly reflect formulation-dependent viscosity and flowability. Increasing polymer concentration from 3% to 4% led to a marked rise in extrusion pressure, consistent with viscosity data. Carbopol inks (C3C, C4C, C3TC, C4TC) required low extrusion pressures of 8–11.6 PSI, while HPMC-based required substantially higher extrusion pressures of 31–40 PSI. This discrepancy can be attributed to their rheological behaviour, as Carbopol dispersions display strong shear-thinning, where the swollen microgel network collapses under shear and flows readily once the yield stress is overcome [57]. However, upon drying, bioinks containing 3% or 4% *w*/*v* Carbopol with the dual drug combination (CBD and THC) were found to be unstable, and CBD-based counterparts exhibited poor structure attributed to their low printability. Barreiro Carpio, Gonzalez Martinez [57] have highlighted in their research that Carbopol inks, although easier to extrude, often exhibited poorer dimensional stability due to their weaker gel framework.

**Figure 4:**
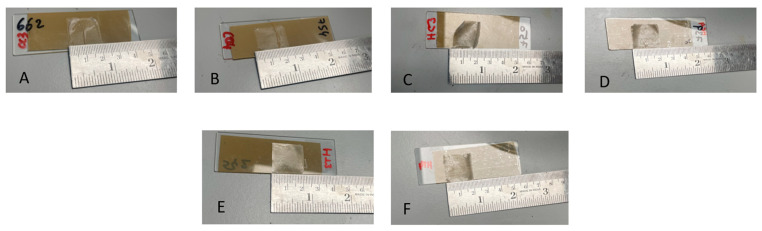
3D-printed buccal cannabinoid films; (**A**) C3C, (**B**) C4C, (**C**) H3C, (**D**) H4C, (**E**) H3TC, and (**F**) H4TC.

As shown in Figure 4, Carbopol-based buccal films (C3C and C4C) failed to retain their shape, whereas HPMC-based films (H3C, H4C, H3TC, and H4TC) maintained their shape and consistency after drying. HPMC polymer has the ability to form a continuous and entangled chain network, which resists alignment within the nozzle, resulting in higher flow resistance and greater extrusion pressures despite lower bulk viscosity values [58]. Notably, the higher extrusion resistance of HPMC was accompanied by superior filament formation and print fidelity, with structures retaining their shape more effectively after deposition.

### 2.4. pH, Drug Content, and Weight of the Buccal Films

Drug content, dried film weight of the 3D printed films was determined, along with the pH of the bioinks. These are shown in Table 5. The pH of the bioinks ranged from 5.99 to 6.80, falling within or close to the physiological salivary pH range (6.2–7.4) [59,60,61], upon adjusting using triethanolamine (TEA) as a neutralizing agent. Drug loading per 200 µL bioink was consistent across formulations. CBD-only systems showed approx. 4 mg CBD content, while dual cannabinoid systems (H3TC, H4TC) exhibited approx. 2 mg CBD and 2 mg THC each. The dried film weights ranged between 16.9 and 27.4 mg depending on polymer type and concentration. Carbopol-based films (C3C, C4C) had higher weights (26–27 mg) compared to HPMC-based films (17–23 mg). This difference can be attributed to Carbopol’s higher water-binding capacity due to the presence of carboxylic groups, which can absorb high volumes of water [62]. This likely enabled Carbopol-based films to retain more residual moisture even after drying, increasing the final weight. An increase in polymer concentration was associated with higher film weight, contributing to the development of more compact and densely printed structures. Similar findings have been reported by Panraksa, Qi [63], who also observed that higher polymer content results in greater solid mass of printed films.

Together, these findings show that both Carbopol and HPMC systems successfully produced films with physiologically acceptable pH, and consistent drug content, for buccal administration.

### 2.5. Mechanical Properties of 3D Printed Films

Mechanical properties (hardness, springiness, and cohesion) of the 3D printed buccal films are represented in Table 6. HPMC films exhibited significantly higher hardness values (6.59–7.38 N) compared to Carbopol films (2.48–4.04 N). The greater hardness of HPMC matrices reflects their stronger polymer chain entanglement and denser film structure after drying, which improves mechanical robustness and handling, while Carbopol films (C3C and C4C) had reduced hardness. The superior mechanical strength of HPMC-based films can be attributed to their inherent capacity to form dense, cohesive matrices during drying, driven by extensive polymer chain entanglement and tighter structural packing [64]. In a study by Peh and Wong [65], incorporation of Carbopol 934P into cellulose-based buccal films was found to reduce their mechanical strength, resulting in the formation of softer, more flexible films. Previous studies have shown that HPMC enhances the mechanical properties of films, with strength increasing in proportion to polymer concentration [64,66]. A similar trend was observed in this study, where raising the HPMC concentration from 3% to 4% led to a general increase in hardness.

Cohesion values reflect the internal bonding strength of the film matrix and its ability to resist structural breakdown under deformation. Cohesion values ranged from 0.824 to 0.999, with HPMC films displaying higher cohesiveness (0.88–1.00) than Carbopol films (0.82–0.90). This suggests that HPMC provides stronger internal bonding within the film matrix, allowing it to resist fracture during handling and buccal adhesion, consistent with previous studies highlighting the role of HPMC in enhancing film cohesiveness [58]. THC-containing formulations (H3TC, H4TC) also demonstrated improved cohesion, likely due to stabilizing interactions of dual cannabinoid–cyclodextrin complexes within the polymer network.

Springiness followed a clear trend, with HPMC films being more elastic (99–100%) compared to Carbopol films (78–82%). Consistent with previous reports, HPMC was generally associated with the formation of more flexible films [58]. Carbopol, being a highly crosslinked poly (acrylic acid) [67], might form a more brittle and rigid structure, limiting its elastic recovery. Overall, HPMC films outperformed Carbopol films in terms of mechanical strength, cohesion, and elasticity, making them more suitable for maintaining integrity during buccal placement and handling.

### 2.6. Mucoadhesive Strength of the Films

**Figure 5:**
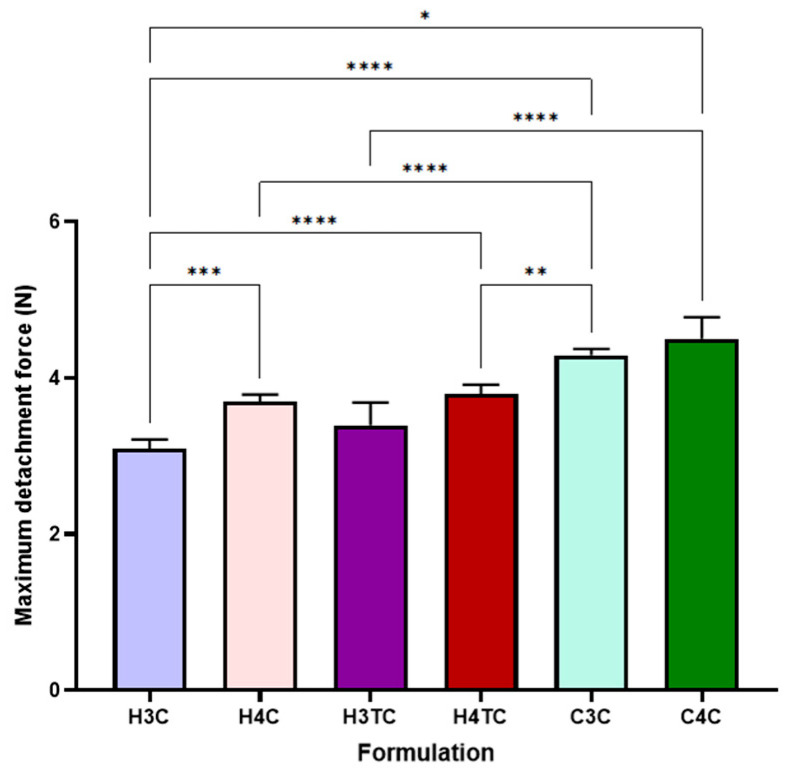
Mucoadhesive strength of 3D printed buccal films of cannabinoids (*n* = 3; mean ± SD). Statistically significant differences are denoted as * *p* < 0.05, ** *p* < 0.01, *** *p* < 0.001 and **** *p* < 0.0001.

The ex vivo mucoadhesion of 3D-printed films, as depicted in Figure 5, reveals significant differences in maximum detachment force (N) across formulations. Carbopol-based films (C3C and C4C) displayed higher mucoadhesiveness than HPMC-based films (H3C, H4C, H3TC and H4TC). The superior mucoadhesive strength of Carbopol-based films (C3C and C4C) compared to HPMC-based films (H3C, H4C, H3TC, H4TC) can be attributed to the chemical and structural properties of Carbopol, a highly crosslinked poly(acrylic acid) polymer. The abundance of carboxyl groups in Carbopol facilitates strong hydrogen bonding and electrostatic interactions with mucin glycoproteins in the mucosal layer, enhancing adhesion despite the films’ weaker mechanical properties, such as brittleness and reduced elasticity. In contrast, HPMC relies primarily on physical entanglement and hydrogen bonding, leading to relatively lower mucoadhesion [68,69].

### 2.7. Swelling Ratio and Dissolution Time

**Figure 6:**
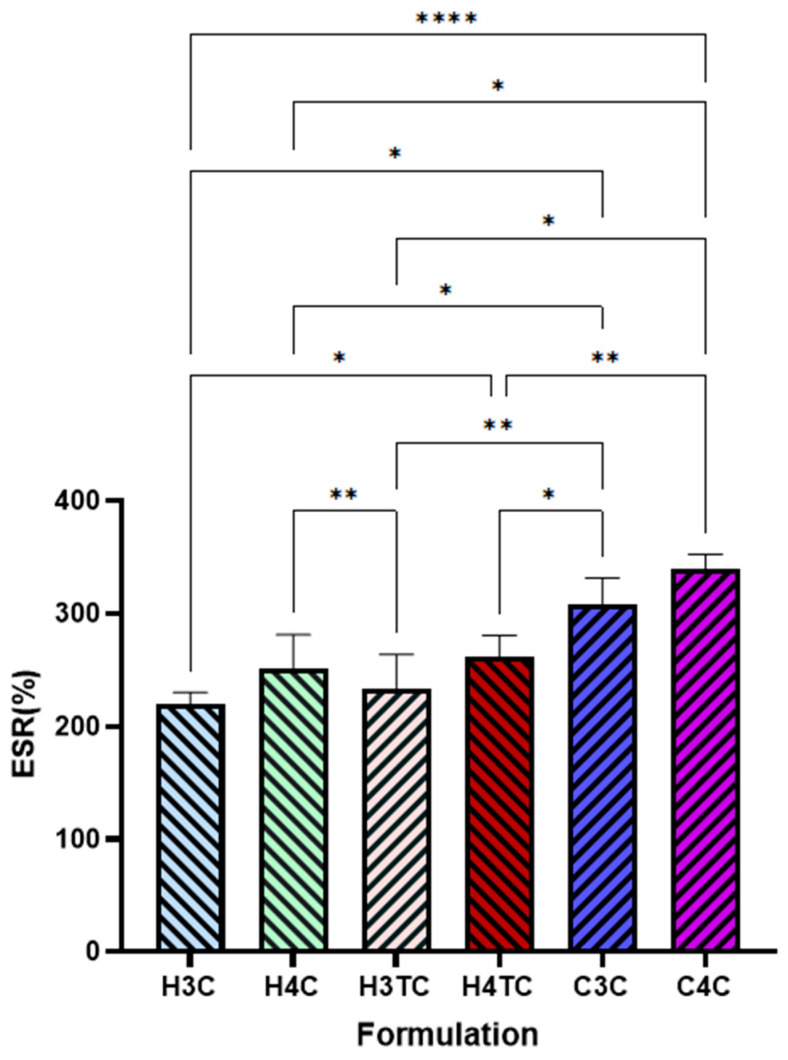
Equilibrium swelling ratio (ESR) % of 3D printed buccal films of cannabinoids (*n* = 3; mean ± SD). Statistically significant differences are denoted as * *p* < 0.05, ** *p* < 0.01 and **** *p* < 0.0001.

The swelling ratio (SR, %) of 3D-printed films is illustrated in Figure 6. HPMC-based films exhibited moderate swelling, with CBD-only formulations (H3C and H4C) and dual-cannabinoid films (H3TC and H4TC) showing slightly higher, though non-significant, values. An increase in polymer concentration in hydroxypropyl methylcellulose (HPMC)-based films led to a higher swelling ratio, with films containing 4% (*w*/*v*) HPMC exhibiting greater swelling than those with 3% (*w*/*v*). This might be due to the hydrophilic nature of HPMC, which contains numerous hydroxyl groups (-OH) that facilitate greater water absorption and matrix expansion. As the HPMC concentration increases, more hydrophilic sites become available for hydrogen bonding with water molecules, enhancing the film’s capacity to uptake fluids and swell [58,70]. In contrast, Carbopol-based films (C3C and C4C) demonstrated significantly greater swelling, which can be attributed to Carbopol’s high water-binding capacity due to its abundant carboxylic groups, allowing it to absorb a substantial volume of water [62].

**Figure 7:**
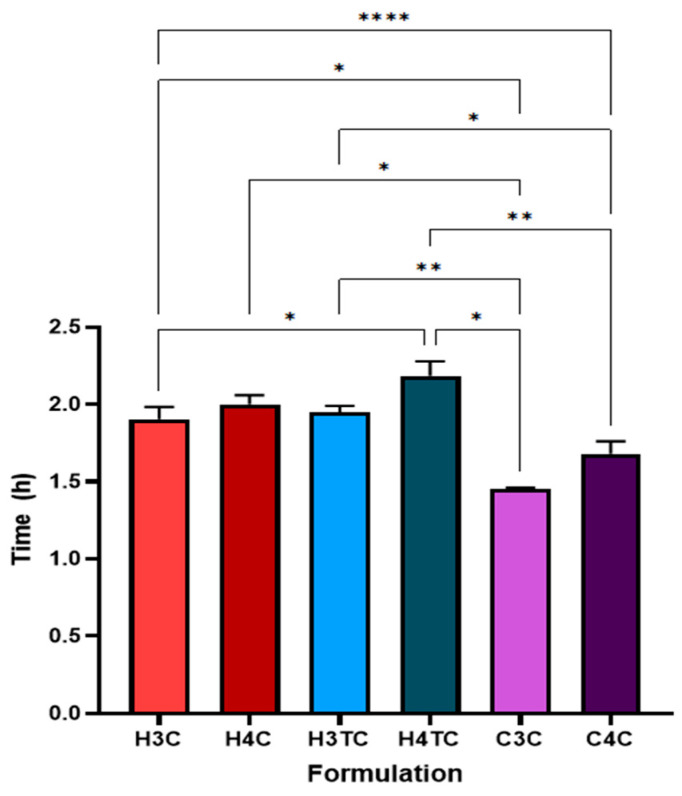
Dissolution time evaluation of cannabinoid loaded buccal films (*n* = 3; mean ± SD). Statistically significant differences are denoted as * *p* < 0.05, ** *p* < 0.01 and **** *p* < 0.0001.

Buccal films incorporated with cannabinoids were also observed for their dissolution time in artificial saliva (Figure 7). HPMC-based films exhibited prolonged dissolution times, with H3C and H4C (CBD-only) formulations dissolving quicker than their dual-cannabinoid counterparts, H3TC and H4TC. The prolonged dissolution times observed in dual-cannabinoid HPMC-based films, despite their higher swelling ratios, can be attributed to enhanced polymer–drug interactions and the role of cyclodextrins. In these dual formulations, both CBD and THC are individually complexed with cyclodextrins, resulting in a higher overall content of these anionic surfactants within the film. Cyclodextrins can form hydrogen bonds with the polymer matrix, strengthening the gel-like network during swelling. This more cohesive network impedes dissolution of the films. Previous research by Bagde and Rohera [71] have reported that anionic surfactants can prolong the dissolution of gels prepared from hydrophilic polymers. In contrast, Carbopol-based films (C3C and C4C) displayed shorter dissolution times. This could be attributed to the high swelling capacity of Carbopol, as noted earlier, which could have accelerated water uptake and structural breakdown. Furthermore, their weaker mechanical properties could have influenced the dissolution of films by reducing their structural integrity and resistance to degradation in aqueous environments.

### 2.8. In Vitro Release of Cannabinoids and Release Kinetics

**Figure 8:**
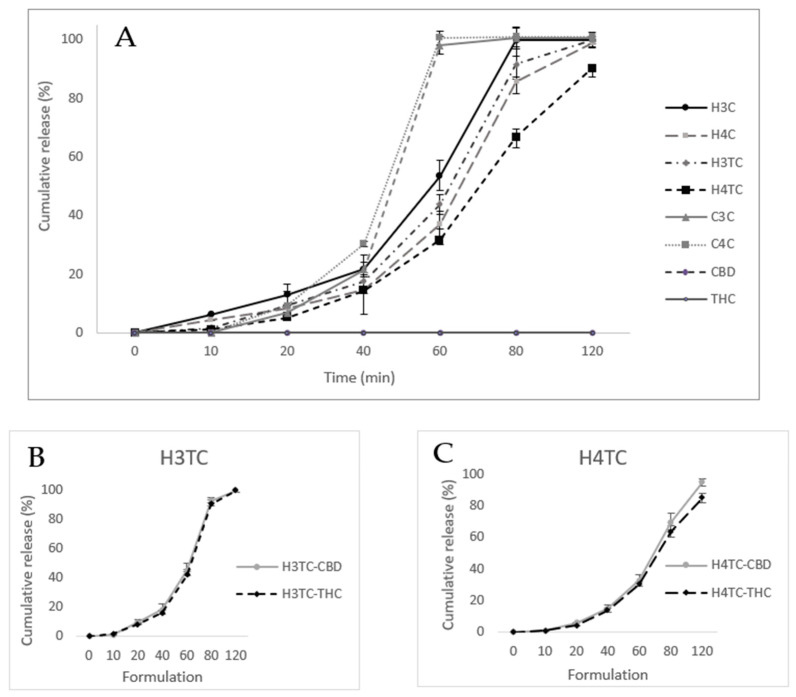
In vitro release profiles of cannabinoid-loaded 3D printed films: (**A**) cumulative release of CBD and THC from all formulations compared with pure drug controls, (**B**) individual release profiles of CBD and THC from film H3TC as a function of time (min), (**C**) individual release profiles of CBD and THC from film H4TC as a function of time (min) (*n* = 3; mean ± SD).

The in vitro release profiles of 3D-printed films are depicted in Figure 8. HPMC-based formulations (H3C, H4C, H3TC, H4TC) demonstrated a slower and more prolonged release of cannabinoids compared to Carbopol-based films (C3C, C4C). This difference can be attributed to the ability of HPMC to form a stable, cohesive gel network upon hydration [58]. Krese, Kovačič [72] demonstrated the influence of HPMC viscosity grade on controlled drug release by evaluating matrices prepared with different grades. Their findings confirmed that viscosity is a key factor in modulating release behaviour, with higher viscosity grades producing a more controlled and sustained release profile. In this study, the use of a high-viscosity grade HPMC (2600–5600 cP) likely contributed to the sustained release of cannabinoids. This is further supported by its superior mechanical strength, as reflected in higher hardness and cohesiveness values. Additionally, the lower swelling ratio observed in HPMC films relative to Carbopol suggests the formation of a denser hydrated matrix, which limited water penetration and slowed the outward diffusion of cannabinoids.

Increasing HPMC concentration from 3% to 4% (H4C, H4TC) further reinforced the gelling network, producing denser matrices and slower release rates. Comparable trends have been reported across different dosage forms, where higher HPMC concentrations consistently resulted in slower drug release [73,74]. Moreover, drug composition significantly influenced release behaviour. Dual-cannabinoid films (H3TC, H4TC) exhibited slower cannabinoid release compared to the CBD-only films (H3C, H4C). This effect may be attributed to the presence of THC, as previous research indicates that, even within inclusion complexes, THC tends to exhibit a slower release profile than CBD [9]. In the present study, film dissolution and cannabinoid release followed parallel trends, reflecting the fact that cannabinoids were dispersed within the polymer matrix rather than existing as separate crystalline phases.

The individual release profiles of CBD and THC from dual-cannabinoid HPMC films (H3TC and H4TC), highlighted in Figure 8B,C, showed comparable patterns, with CBD exhibiting a slightly higher release than THC.

Overall, Carbopol films exhibited faster release, approx. 100% in 60 min, owing to extensive swelling and weaker matrix stability, whereas HPMC films provided a more sustained cannabinoid diffusion.

The release kinetics of cannabinoids from HPMC- and Carbopol-based buccal films were analyzed using multiple kinetic models, and the corresponding determination coefficient (R^2^) are summarized in Table 7. For HPMC-based formulations (H4C, H4C, H3TC, AND H4TC), Korsmeyer–Peppas analysis showed the highest determination coefficient (R^2^ = 0.952–0.992), suggesting that drug release occurs via a non-Fickian mechanism, driven by both diffusion through the hydrated HPMC matrix and polymer chain relaxation/swelling. This dual mechanism is well documented in the literature, as HPMC undergoes swelling upon contact with aqueous media to form a viscous gel layer that modulates diffusion [75]. Previous studies on HPMC-based films have similarly demonstrated that their drug release follows the Korsmeyer–Peppas model [76,77,78]. Furthermore, zero-order modelling yielded high correlation coefficients (R^2^ = 0.888–0.962), indicating that drug release from HPMC films follows a near-constant release profiles. The Hixson–Crowell model also provided good fits (R^2^ = 0.886–0.940), particularly for THC-containing films, indicating that matrix erosion contributes alongside diffusion [79].

Unlike HPMC-based films, Carbopol formulations showed weaker zero-order fits (R^2^ ≈ 0.77) and lower Korsmeyer–Peppas R^2^ values (around 0.64). Instead, they aligned strongly with the Hixson–Crowell model (R^2^ = 0.926–0.928), suggesting that matrix erosion was the dominant mechanism of release [79]. The burst-type release seen experimentally is therefore consistent with an erosion-driven mechanism.

### 2.9. FTIR

**Figure 9:**
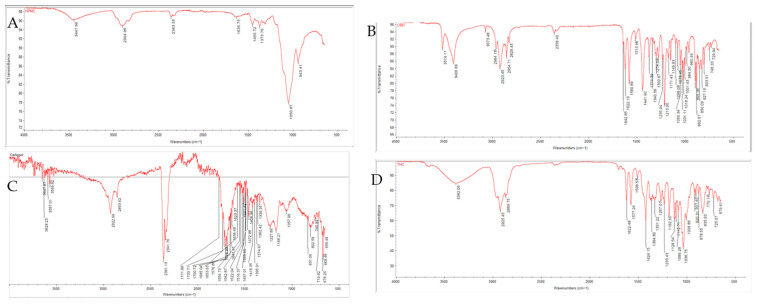
FTIR spectra of (**A**) HPMC, (**B**) CBD, (**C**) Carbopol, and (**D**) THC.

**Figure 10:**
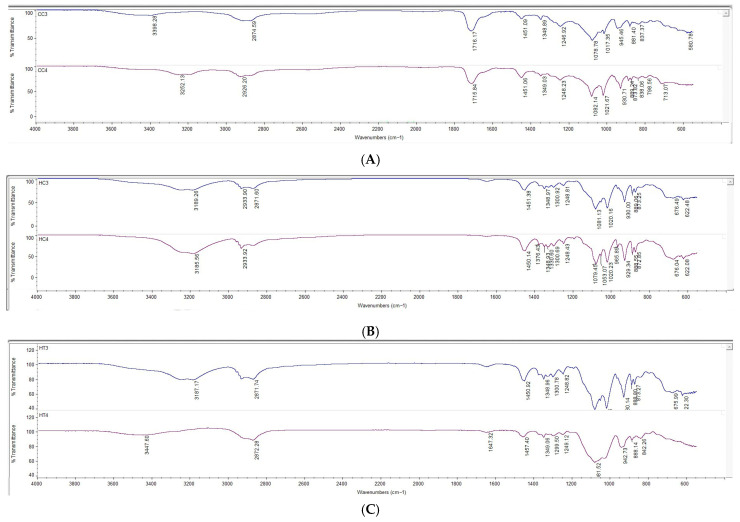
FTIR spectra of 3D printed buccal films; (**A**) C3C and C4C, (**B**) H3C and H4C, and (**C**) H3TC and H4TC.

The FTIR spectra of the polymers (HPMC and Carbopol) and cannabinoids (CBD and THC) are depicted in Figure 9. The FTIR spectra of the 3D-printed buccal films, as shown in Figure 10, reveal characteristic absorption bands across the formulations in transmittance mode from 4000 to 600 cm^−1^.

HPMC films (Figure 10B,C) exhibited broad O–H stretching vibrations at 3417–3446 cm^−1^, indicative of extensive hydrogen bonding from hydroxyl groups in the polymer [80,81,82]. Characteristic fingerprint region bands at 2870–2935 cm^−1^ (C–H stretching), 1369–1370 cm^−1^ (O–H bending), and 1050–1100 cm^−1^ (C–O stretching) [83]. The absence of significant shifts in C–O and other cellulose-related bands indicate homogeneous integration of the complexes without altering the polymer backbone.

Prominent bands around 2850 and 2950 cm^−1^ correspond to C–H stretching vibrations of the CBD–HP-β-CD and THC–HP-β-CD inclusion complexes. The absorption around 1031 cm^−1^ is assigned to C–O and C–O–C stretching vibrations within the cyclodextrin framework. In dual cannabinoid films, peaks for conjugated carbonyl groups were observed around 1647 cm^−1^, which may reflect contributions from both CBD and THC inclusion complexes [10,84,85].

Carbopol films (Figure 10A) displayed broad O–H stretches around 3468–3473 cm^−1^ and C–H stretches at 2936–2938 cm^−1^. A prominent carbonyl (C=O) band around 1716–1717 cm^−1^ confirmed the polyacrylic acid backbone, essential for maintaining acidic functionality that facilitates mucoadhesion via electrostatic interactions with mucosal surfaces. Additional bands at 1450–1457 cm^−1^, 1240–1248 cm^−1^, and fingerprint peaks at 1178 and 1078 cm^−1^ were consistent with polymer-specific vibrations [86,87]. The spectra revealed no significant shifts or disappearance of characteristic bands, indicating compatibility between Carbopol and cannabinoid complexes, with preservation of structural integrity during 3D printing and drying. No unexpected peaks or disappearances were observed, demonstrating excellent compatibility between cannabinoids in inclusion complexes and polymers.

### 2.10. SEM

**Figure 11:**
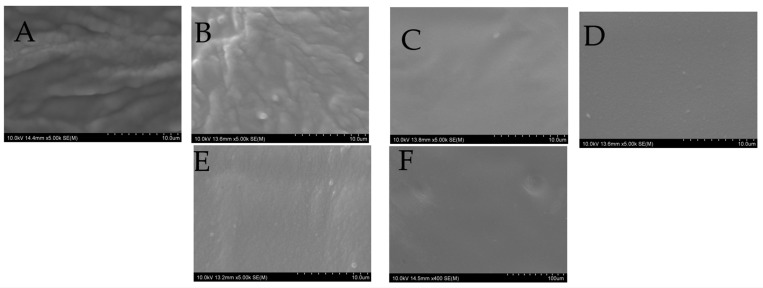
SEM of 3d printed buccal films (**A**) C3C, (**B**) C4C, (**C**) H3C, (**D**) H4C, (**E**) H3TC, AND (**F**) H4TC.

Scanning electron microscopy (SEM) images in Figure 11 illustrate the surface morphologies of the 3D-printed buccal films. Carbopol-based films (C3C, C4C) display rough, wavy surfaces with visible irregularities, indicative of a heterogeneous structure. The rough and wavy surface morphology observed in Carbopol-based films (C3C and C4C) can be attributed to the highly crosslinked poly(acrylic acid) structure of Carbopol, which promotes uneven shrinkage and pore formation during drying, resulting in a brittle and heterogeneous matrix [67].

In contrast, HPMC-based films (H3C, H4C, H3TC, and H4TC) exhibit smoother, more uniform surfaces. The smoother surfaces of HPMC-based films might be due to the flexible, linear cellulose backbone of the polymer, which facilitates denser packing and homogeneous film formation through extensive hydrogen bonding [64]. SEM observations from prior studies indicate that HPMC films maintain a uniform and smooth surface structure [88,89].

The smoother, non-porous surface of HPMC-based films correlates with the observed sustained drug release, as it limits rapid water penetration and reduces initial burst release. In contrast, the porous and less structured morphology of Carbopol films facilitates faster hydration and erosion-driven release.

### 2.11. Ex Vivo Penetration of Cannabinoids from Printed Films

**Figure 12:**
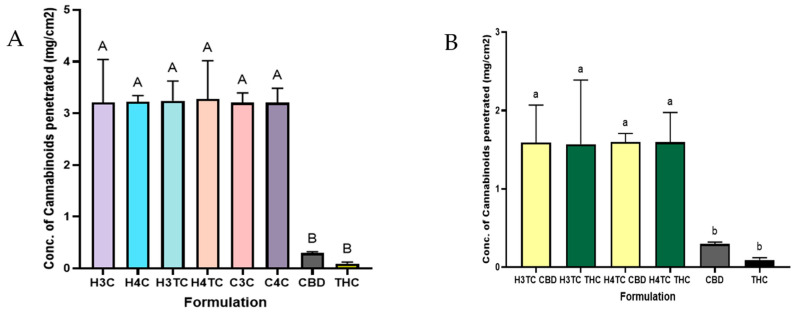
Ex vivo penetration profiles of (**A**) Total cannabinoids from 3d printed films, (**B**) Individual permeation profiles of CBD and THC compared to pure CBD and THC distillate. Data represents mean ± SD (*n* = 3). Different letters indicate statistically significant differences between groups at *p* < 0.05. Values sharing at least one common letter are not significantly different.

The ex vivo penetration profiles demonstrated that all 3D-printed films markedly enhanced cannabinoid transport across the buccal mucosa compared with pure CBD and THC. As shown in Figure 12A, both HPMC- and Carbopol-based films (H3C, H4C, H3TC, H4TC, C3C, C4C) achieved significantly higher penetration concentrations (around 3.0–3.3 mg/cm^2^) than pure cannabinoids (<0.5 mg/cm^2^). Statistical grouping confirmed that all polymeric formulations belonged to the same significance group (A), while unformulated cannabinoids were significantly lower (B). This highlights the critical role of polymeric carriers in improving cannabinoid solubility and permeability. Since the films dissolved within approximately 2 h as observed from their dissolution time, the residence and release periods were comparable across formulations, which explains why cannabinoid penetration into the buccal mucosa was similar for both HPMC- and Carbopol-based films.

The enhanced permeation observed with the 3D-printed films can be explained by several complementary mechanisms. Firstly, the incorporation of a terpene combination likely played a major role, as terpenes have been widely reported to act as natural permeation enhancers by interacting with the lipid domains of the buccal mucosa and thereby improving cannabinoid penetration. This is consistent with previous findings with earlier reports that terpenes such as limonene and menthol enhance the buccal absorption of lipophilic drugs, including cannabinoids [46,47]. Furthermore, the mucoadhesive nature of HPMC and Carbopol prolonged the residence time of the films at the buccal surface, increasing mucosa contact period of the drug. This agrees with prior work showing that bioadhesive polymers enhance transmucosal delivery by preventing premature clearance and improving drug retention at the site of absorption [90,91]. Together, these factors explain the significantly greater cannabinoid transport achieved with the 3D-printed films compared to unformulated CBD and THC.

Figure 12B, further compared CBD and THC permeation from dual-cannabinoid HPMC films (H3TC, H4TC). Both cannabinoids achieved comparable permeation (around 1.5–1.6 mg/cm^2^), significantly higher than their pure cannabinoid counterparts. No significant differences were observed between CBD and THC permeation within the same formulation.

Overall, these results demonstrate that 3D-printed buccal films dissolve within a clinically relevant timeframe and provide consistent, enhanced transmucosal delivery of cannabinoids compared with unformulated drugs.

## 3. Conclusions

This study successfully developed and characterized 3D-printed buccal films using HPMC and Carbopol polymers for cannabinoid delivery, demonstrating their potential as effective transmucosal drug delivery systems. These were incorporated with a specific terpene blend of 3.125% 1,8-cineole, 0.625% d-limonene, 0.625% L-menthol, and 0.625% α-pinene, which outperforming all other terpene combinations, and the synthetic enhancer Azone in ex vivo penetration studies with CBD. Rheological assessment showed that polymer concentration and drug composition significantly influenced bioink viscosity, with Carbopol exhibiting higher viscosity but easier extrusion due to shear-thinning, whereas HPMC required higher extrusion pressures yet produced superior filament formation and print fidelity. All formulations maintained physiologically compatible pH and consistent cannabinoid content.

Mechanical testing revealed that HPMC-based films possessed greater hardness, cohesion, and elasticity, whereas Carbopol films, although softer and more brittle, displayed higher mucoadhesive strength through carboxyl group-mediated hydrogen bonding with mucin. Swelling and dissolution studies indicated that Carbopol films absorbed more water and dissolved rapidly, whereas HPMC films, particularly dual-cannabinoid formulations, exhibited moderated swelling and prolonged dissolution due to cohesive polymer–drug–cyclodextrin networks.

In vitro release studies confirmed that HPMC films provided sustained cannabinoid release, with dual-cannabinoid systems releasing more slowly than CBD-only films. Carbopol films showed rapid release consistent with their higher swelling and weaker matrix stability. Kinetic analysis revealed that HPMC films followed a non-Fickian release mechanism, reflecting combined diffusion and polymer relaxation/swelling, whereas Carbopol films aligned with the Hixson–Crowell model, indicating matrix erosion-driven, burst-type release.

FTIR analysis confirmed compatibility between cannabinoids in inclusion complexes and both polymers, with no significant shifts or disappearance of characteristic bands, while SEM imaging revealed smooth, uniform surfaces in HPMC films and rough, heterogeneous surfaces in Carbopol films, reflecting differences in polymer chain flexibility and crosslinking. Ex vivo penetration studies further demonstrated that all 3D-printed films markedly enhanced cannabinoid transport across the buccal mucosa compared with unformulated CBD and THC.

Overall, HPMC-based films offered superior mechanical strength, structural integrity, controlled swelling, and sustained release, making them ideal for effective buccal delivery, whereas Carbopol films provided enhanced mucoadhesion but were limited by rapid dissolution and weaker mechanical properties. Although combining HPMC and Carbopol could potentially provide synergistic mechanical and mucoadhesive properties, this study intentionally evaluated them separately to clearly distinguish their individual contributions to printability, mechanical behaviour, and drug release. These findings highlight the critical role of polymer type, concentration, and drug composition in tailoring the physicochemical, mechanical, mucoadhesive, and release properties of 3D-printed cannabinoid buccal films, establishing a solid basis for the development of optimized transmucosal drug delivery systems.

## 4. Materials and Methods

Hydroxypropyl methyl cellulose (HPMC) was purchased from Sigma-Aldrich, Auckland, New Zealand. Carbopol 934P was purchased from DKSH LabShop, Auckland, New Zealand, D-Limonene from Thermo Fisher, New Zealand, 1,8-cineole, α-Pinene from Sigma-Aldrich, New Zealand, and l-Menthol from Chem express supplied by Focus Bioscience, Brisbane, Australia. Laurocapram (Azone®) 95% was purchased from AK Scientific, Inc., Union City, CA, USA. Medium-chain triglyceride (MCT) oil was purchased from local commercial supplier, Auckland, New Zealand. Phosphate-buffered saline (PBS) tablets (pH 7.4), Poly-ethylene glycol (PEG 400), HP-β-CD, and acetonitrile were purchased from Sigma-Aldrich, New Zealand. Pure isolated CBD and distilled oil of THC were provided by Helius Therapeutics Ltd., Auckland, New Zealand. All other reagents used were of analytic grade.

Artificial/stimulated saliva was prepared by dissolving 1.5% *w*/*v* potassium chloride (KCl), 0.43% *w*/*v* sodium chloride (NaCl), 0.22% *w*/*v* calcium chloride (CaCl_2_), 0.42% *w*/*v* sodium bicarbonate (NaHCO_3_), and an appropriate amount of sodium dihydrogen phosphate monohydrate (NaH_2_PO_4_·H_2_O) in distilled water [92].

### 4.1. Ex Vivo Penetration Studies of CBD with Terpenes

#### 4.1.1. Porcine Buccal Mucosa Preparation

Fresh pig heads were purchased from a local butcher shop, Auckland, New Zealand. Within an hour of slaughter, inner regions of the cheek were excised carefully uniformly below 0.5 mm thickness with a scalpel and rinsed with saline

**Figure 13:**
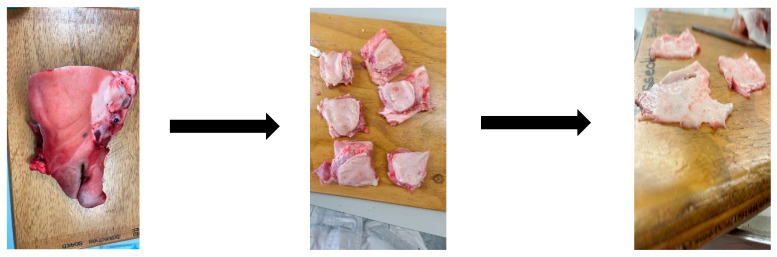
Stepwise preparation of buccal porcine mucosa tissue showing (from **left** to **right**): Freshly obtained porcine cheek tissue, sectioning into smaller pieces, and removal of underlying connective and adipose layers to isolate the mucosal membrane for experimental use.

Figure 13 below, highlights the porcine buccal mucosa excised. All experiments were performed with porcine mucosa obtained from three different pigs.

If required, the tissues were frozen in a standard freezer (−15 °C to −25 °C) in freshly prepared PBS with 2% DMSO or 10% BSA, added as cryoprotectants [93].

#### 4.1.2. Preparation of Cannabinoid Solutions with Permeation Enhancers

Donor solutions were prepared by mixing CBD (25 mg/mL) in MCT oil, to which different concentrations and combinations of the terpenes d-limonene, l-menthol, α-pinene, and 1,8-cineole or synthetic enhancer laurocapram (Azone®) were added as listed in Table 8 below.

#### 4.1.3. Ex Vivo Penetration with Franz Cells

**Figure 14:**
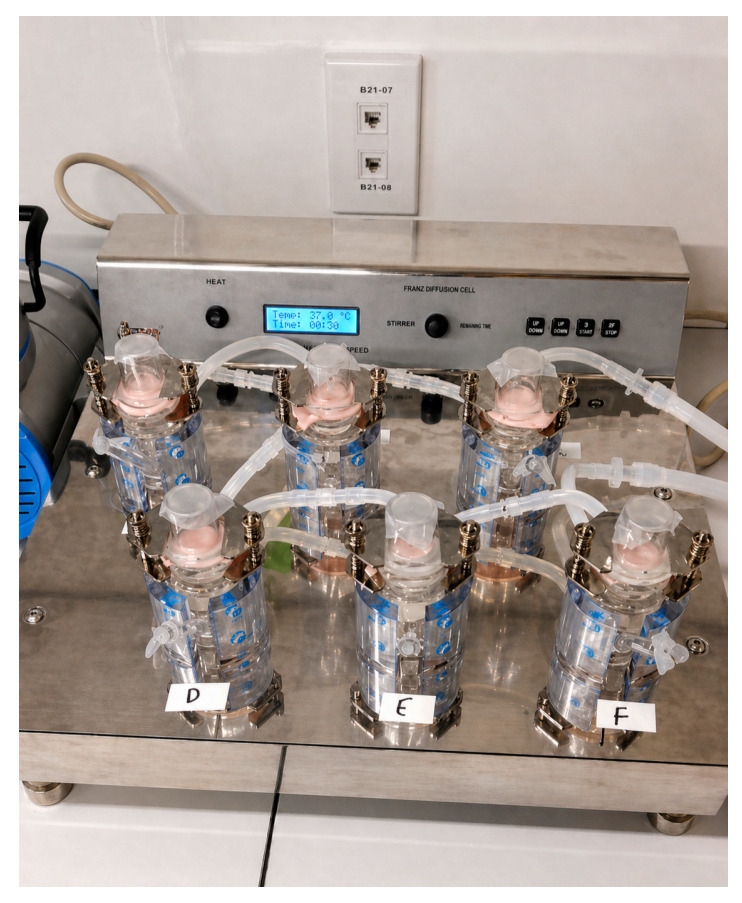
Setup of the ex vivo penetration using Franz cell.

The receptors were filled with 20 mL of phosphate-buffered saline (PBS) containing 3% Tween-20 while maintaining sink conditions, and the cells were pre-warmed to 37 °C for one hour to ensure proper temperature equilibration. A piece of porcine buccal mucosa, carefully cut to match the diameter of the cell compartments, was positioned between the donor and receptor sections and securely clamped in place to create a stable barrier. Subsequently, 1 mL of the prepared donor solution was introduced into the corresponding donor chamber, the openings were sealed with parafilm to prevent any solvent evaporation during the course of the experiment, and it was set up as highlighted in Figure 14. After a two-hour period, the porcine buccal mucosa was carefully removed from the setup. The tissue was then gently wiped with absorbent tissue to remove any residual donor solution from the surface of the mucosa. Following this, the CBD that had penetrated through the mucosal layers was extracted for further analysis. This experiment was done with triplicates of each of the above prepared solutions.

#### 4.1.4. Extracting the Cannabinoids Deposited in the Mucosa

The porcine buccal mucosa, after permeation, was washed thrice with deionized water, following which it was cut into small pieces. The mucosa was frozen with liquid nitrogen and ground into a powder. A homogenizer (IKA T25 ULTRA-TURRAX, Staufen im Breisgau, Germany) was used at 8000 rpm for 5 min to homogenize the powder dissolved in acetonitrile/water (1:1 *v*/*v*) solution. The homogenized solution was then centrifuged at 4000 rpm for 20 min, supernatant filtered, and filtrate analyzed by HPLC (HPLC–UV; Shimadzu Corporation, Kyoto, Japan). The penetration was calculated by determining the amount of CBD per surface area (µg/cm^2^) [49].

### 4.2. Preparation of Mucoadhesive Bioinks

HPMC or Carbopol (1–5% *w*/*v*) were weighed and gradually dispersed in distilled water under continuous magnetic stirring at room temperature to prevent clumping, forming a homogenous polymeric hydrogel solution. To this, polyethylene glycol 400 (PEG 400) was added as the plasticizer at 5/10% *v*/*v*. Cannabinoids, either CBD alone or a combination of CBD and THC in the form of inclusion complexes with HP-β-CD, were introduced into the solution at a final concentration of 20 mg/mL under gentle stirring. Then Mannitol (200 mg) was incorporated as a sweetener, and terpenes (D-limonene, L-menthol, α-pinene, and 1,8-cineole) were added as permeation enhancers. The complete mixture was homogenized using a magnetic stirrer for 30–45 min until uniform bioinks were obtained. The prepared bioinks were loaded into syringe cartridges for immediate printing or stored at 4 °C for further use. The procedure was developed based on modifying the methods reported in previous studies involving either cannabinoids or the fabrication of buccal films using mucoadhesive polymers [22,94]. The pH of the bioinks was adjusted to 6.4 using triethanolamine. The composition and concentration of the bioink components of cannabinoid-loaded mucoadhesive buccal films are summarized in Table 9.

#### Viscosity of Bioinks

The viscosity of the prepared bioinks was measured using a Brookfield RST-SST rheometer fitted with a rotating ramp measuring spindle. Viscosity values were recorded at a constant shear rate of 100 s^−1^ to assess the flow properties and suitability of the bioinks for extrusion-based 3D bioprinting.

### 4.3. Bioprinting Mucoadhesive Films of Cannabinoids

The design of the buccal films was created using SolidWorks software 2023, establishing a film model with dimensions of 20 × 20 × 0.35 mm. The 3D computer-aided design (CAD) model was exported in STL file format and uploaded to Slice3r software version 3, which converted the design into a layer-by-layer instructional format (G-code) required for printing. Repetier-Host software version 2.3.2 was employed to fine-tune printing parameters, including extrusion pressure and printing speed, ensuring optimal deposition of the bioink. The generated G-code from Slice3r was used by the Allevi 3D bioprinter to fabricate the buccal films. Printing was performed on individual glass slides covered with adhesive tape to facilitate easy detachment post-printing. The bioinks were extruded using a previously optimized 25 G needle, selected based on the flow and rheological properties of the formulations. Following printing, all films were air-dried in the dark for 48 h to achieve structural stability. The dried films were subsequently stored in a dark environment for further evaluations.

### 4.4. Determining pH, Drug Content, and Weight of Printed Films

The pH of the developed bioinks was measured using a calibrated bench-top pH metre (Interlab, Auckland, New Zealand) to ensure compatibility of printed films with the buccal mucosa. The drug content of the films was quantified, employing high-performance liquid chromatography (HPLC) to confirm accurate and uniform incorporation of the active cannabinoid compounds. Printed films were weighed using an electronic balance.

### 4.5. Mechanical Properties of the Films

Mechanical properties of the films were determined using a texture analyser (Stable Micro Systems, Godalming, UK) fitted with a 50 N load cell. Printed films were placed in individual wells of a 12-well plate and positioned on the analyser platform. A stainless-steel spherical probe (2 mm diameter) was used to compress each film to a depth of 1 mm at a test speed of 0.8 mm·s^−1^, with a 5 s relaxation period between two compression cycles (n = 3). The results were analyzed using TA.XT Exponent software version 6.2 to quantify the mechanical parameters. The procedure was adapted from a previously employed method by De Souza Ferreira, Da Silva [95].

### 4.6. Mucoadhesive Strength of Cannabinoid Loaded Films

The mucoadhesive strength of the optimized 3D printed buccal films was assessed using freshly excised porcine buccal mucosa. A texture analyser (Stable Micro Systems, UK) was employed to measure the force required to detach the films from the mucosal surface, providing a quantitative assessment of their adhesion. The excised mucosal tissue was trimmed to a suitable size and firmly attached to the upper probe of the texture analyzer using double-sided adhesive tape to ensure stable contact throughout the test. Printed films were placed in individual wells of a 12-well plate and positioned on the analyser platform, below the probe. The probe was then lowered until it contacted the printed surface and maintained a constant contact pressure for 60 s to allow adhesive bonding between the film and the mucosa. After this contact period, the probe was withdrawn vertically at a speed of 0.8 mm·s^−1^, and the maximum detachment force (N) required to separate the probe (with attached mucosa) from the gel was recorded. Each experiment was conducted in triplicate (*n* = 3), with temperatures maintained at 37 °C. The mean peak force value was used as an indicator of the mucoadhesive strength of the formulation.

### 4.7. Swelling Ratio and Dissolution Time of Printed Films

The 3D printed buccal films were first weighed (W_1_) and placed inside plastic tissue cassettes. The cassettes were then immersed in simulated saliva at 37 °C for 1 h. After incubation, the films were carefully removed, gently blotted with tissue paper to remove excess surface fluid and weighed again using a digital balance (W_2_). The degree of swelling, representing the fractional increase in weight of the films due to saliva absorption, known as the swelling ratio, was calculated using Equation (1) [96].

(1) $$Swelling\;ratio=\frac{W2-W1}{W1}\times 100$$

The dissolution time of the 3D printed buccal films was evaluated using simulated saliva (pH 6.8) at 37 ± 0.5 °C to mimic physiological conditions. A single film was carefully placed in a Petri dish containing 10 mL of the medium. The film was observed continuously for signs of softening, swelling, disintegration, or complete dissolution. Gentle agitation was applied intermittently to simulate oral movements. The time taken from initial contact with the medium until complete disintegration or dissolution of the film was recorded as the dissolution time. Each formulation was tested in triplicate, and the average dissolution time was calculated [97].

### 4.8. In Vitro Drug Release and Drug Release Kinetics

In vitro release studies were conducted using a Franz diffusion cell apparatus (Orchid Scientific, Nashik, India) equipped with dialysis membranes (MWCO 14,000 Da) to evaluate cannabinoid release from optimized formulations over a 4 h period. Prior to use, the membranes were hydrated for 24 h. The Franz cells were maintained at 37 °C and allowed to equilibrate for one hour before the experiment commenced. Each receptor chamber was filled with 20 mL of phosphate-buffered saline (PBS) containing 3% Tween 20, which was continuously stirred at 300 rpm. The pre-soaked cellulose membrane was then placed between the donor and receptor compartments and securely clamped. Subsequently, the printed buccal formulations were placed in the donor chamber, and all openings were sealed with parafilm to minimize evaporation. At specified time intervals of 5, 10, 15, 30, 60, 120, and 240 min, 1 mL samples were withdrawn from the receptor chamber and immediately replaced with fresh medium pre-warmed to 37 °C. The inclusion of 3% Tween 20 and periodic replacement of withdrawn samples with fresh pre-warmed medium ensured that the drug concentration remained below 10% of its saturation solubility at all times, thereby maintaining sink conditions. The collected samples were analyzed using HPLC to quantify the amount of cannabinoid released. All experiments were performed in triplicate for each optimized formulation.

Release kinetics of CBD and THC were analyzed using KinetDS software 3.0 (rev. 2010). To characterize the drug release mechanisms from the formulations, mathematical models applied included Zero-order, First-order, Higuchi, Korsmeyer-Peppas, and Hixson-Crowell models.

### 4.9. FTIR

FTIR analysis of the optimized 3D printed buccal films was performed using a Thermo Scientific FTIR spectrometer (Nicolet iS10, Madison, WI, USA). The films were analyzed to evaluate possible interactions between cannabinoids in inclusion complexes and mucoadhesive polymers. Samples were placed directly onto a monolithic diamond crystal cell, and spectra were collected over the wavenumber range of 4000–500 cm^−1^. Each spectrum was obtained at a resolution of 4 cm^−1^ with 32 scans to ensure accuracy and reproducibility. A background spectrum was recorded using a clean, empty cell at room temperature (25 ± 2 °C) and served as a reference.

### 4.10. SEM

The surface morphology of the 3D printed films was examined using SEM. Samples were coated with a thin layer of platinum using an ion sputter coater (Hitachi E-1045, Tokyo, Japan) and imaged with a Schottky field emission scanning electron microscope (Thermo Scientific, HITACHI SU-70).

### 4.11. Ex Vivo Penetration of Cannabinoids from Buccal Films

Ex vivo permeation of cannabinoids from the 3D printed films was evaluated using freshly excised porcine buccal mucosa mounted on a Franz diffusion cell apparatus (Orchid Scientific, India). The procedure followed the methodology outlined previously, allowing assessment of drug penetration across the buccal mucosa.

## 5. Statistical Analysis

Experimental data were processed and analyzed using R programming and GraphPad Prism software (version 10.2.3). For datasets meeting the assumptions of normality and homogeneity of variances, statistical significance was determined using one-way analysis of variance (ANOVA). In cases where these assumptions were violated, the non-parametric Kruskal–Wallis test was employed. When significant differences were detected, post hoc comparisons were conducted using Tukey’s multiple comparison test. A probability value of *p* < 0.05 was considered indicative of statistical significance.

## Figures and Tables

**Table 2:** Composition of 3D printing inks with different mucoadhesive polymers and cannabinoid inclusion complexes.

Code	Mucoadhesive Polymer	Drug in Inclusion Complex
H3C	3% HPMC	CBD (20 mg/mL)
H4C	4% HPMC	CBD (20 mg/mL)
H3TC	3% HPMC	THC + CBD (1:1, 20 mg/mL total)
H4TC	4% HPMC	THC + CBD (1:1, 20 mg/mL total)
C3C	3% Carbopol	CBD (20 mg/mL)
C4C	4% Carbopol	CBD (20 mg/mL)
C3TC	3% Carbopol	THC + CBD (1:1, 20 mg/mL total)
C4TC	4% Carbopol	THC + CBD (1:1, 20 mg/mL total)

**Table 3:** Optimization of PEG concentration for extrusion-based 3D printing of buccal films (*n* = 3; mean ± SD).

Ink Composition	Temperature (°C)	Nozzle	Speed (mm·s^−1^)	Pressure (PSI)	Observations
**HPMC 4% + PEG 10%**	21.9–21.0	25G	6	24.6 ± 0.3	Acceptable print fidelity (thin). Films sticky upon drying due to excess PEG.
**HPMC 4% + PEG 5%**	20.0–19.0	25G	6	37.8 ± 0.2	Optimal print quality (smooth, uniform lines). Stable films post-drying.

**Table 4:** Printing parameters of different ink compositions, including temperature range, nozzle size, printing speed, and applied pressure during material extrusion 3D printing (*n* = 3; mean ± SD).

Ink Composition	Temperature (°C)	Nozzle	Speed (mm·s^−1^)	Pressure (PSI)
C3C	21.5–22.5	25G	6	10.1 ± 0.1
C4C	21.4–22.9	25G	6	11.6 ± 0.7
C3TC	21.3–22.8	25G	6	8 ± 0.6
C4TC	21.9–23.8	25G	6	9.9 ± 0.9
H3C	21.5–23.6	25G	6	31.4 ± 0.5
H4C	20.4–21.3	25G	6	39.7 ± 0.7
H3TC	21.4–21.7	25G	6	33.1 ± 0.8
H4TC	21.9	25G	6	40 ± 0.2

**Table 5:** pH, drug content, and weight of dried 3D-printed buccal cannabinoid films (*n* = 3; mean ± SD).

Ink Composition	pH	Drug Content (per 200 µL)	Weight of Dried Films (mg)
C3C	6.57 ± 0.4	4.1 ± 1.9 mg CBD	26.2 ± 0.3
C4C	6.34 ± 0.2	4.0 ± 1.1 mg CBD	27.4 ± 0.5
H3C	6.80 ± 0.01	4.1 ± 0.7 mg CBD	16.9 ± 0.8
H4C	6.40 ± 0.04	4.01 ± 0.03 mg CBD	22.8 ± 0.1
H3TC	6.10 ± 0.3	2.0 ± 0.8 mg CBD; 2.1 ± 0.3 mg THC	20.9 ± 0.9
H4TC	5.99 ± 0.07	2.0 ± 0.6 mg CBD; 2.0 ± 0.1 mg THC	26.5 ± 0.2

**Table 6:** Texture profile analysis of 3D-printed buccal cannabinoid films showing hardness, cohesion, and springiness (*n* = 3; mean ± SD). Different superscript letters within the same column indicate statistically significant differences between groups at *p* < 0.05. Values sharing at least one common superscript letter are not significantly different.

Formulation	Hardness (N)	Cohesion	Springiness (%)
**H3C**	6.9 ± 0.02 ^ab^	0.98 ± 0.03 ^a^	100.00 ± 0.1 ^ab^
**H4C**	7.2 ± 0.01 ^a^^c^	0.88 ± 0.03 ^a^	99.42 ± 1.4 ^a^
**H3TC**	6.6 ± 0.02 ^a^^c^	0.97 ± 0.08 ^ab^	99.98 ± 1.0 ^b^
**H4TC**	7.4 ± 0.05 ^b^^d^	0.99 ± 0.03 ^b^^c^	99.28 ± 0.8 ^c^
**C3C**	2.5 ± 0.05 ^c^^d^^e^	0.82 ± 0.06 ^b^^c^	81.53 ± 0.2 ^c^
**C4C**	4.0 ± 0.08 ^e^	0.90 ± 0.04 ^c^	78.67 ± 0.9 ^d^

**Table 7:** Kinetic modelling of cannabinoid release from 3D printed buccal films. Shaded cells indicate the model providing the best fit to the release data, as determined by the highest coefficient of determination (R_2_). Peach shading represents formulations best fitted by the Korsmeyer–Peppas model, while green shading represents formulations best fitted by the Hixson–Crowell model.

Formulations	Zero-Order	First Order	Higuchi	Korsmeyer-Peppas	Hixon-Crowell
	R^2^				
H3C	0.8879	0.8618	0.3605	0.9581	0.8899
H4C	0.9154	0.9037	0.2274	0.9517	0.9288
H3TC CBD	0.9245	0.7675	0.0718	0.9575	0.8862
H3TC THC	0.9160	0.8317	0.1287	0.9736	0.9065
H4TC CBD	0.9614	0.8335	0.0509	0.9857	0.9402
H4TC THC	0.9623	0.8411	0.0266	0.9917	0.9382
C3C	0.7717	0.3670	−1.3985	0.6478	0.9284
C4C	0.7728	0.3557	−1.5885	0.6360	0.9257

**Table 8:** Permeation enhancers for ex vivo penetration of CBD.

Solution Code	Concentration of Permeation Enhancer(s) Added (µg/mL)
CBD	nil
C5l	CBD + 5% limonene
C5M	CBD + 5% Menthol
C5P	CBD + 5% Pinene Conc. (µg/mL)
C5C	CBD + 5% Cineole (µg/mL)
C5A	CBD + 5% Azone (µg/mL)
C1.25MCLP	CBD + 1.25%mclp (µg/mL)
C3.12L0.6MCP	CBD+3.125%L, 0.625%MCP (µg/mL)
C2.5LC	CBD+2.5%L, 2.5%C (µg/mL)
C2.5MP	CBD+2.5%M, 2.5% P(µg/mL)
C3.12M0.62LCP	CBD+3.125%M, 0.625%LCP (µg/mL)
C3.12P0.62LCM	CBD+3.125%P, 0.625%LCM (µg/mL)
C2.5MC	CBD+2.5%M, 2.5%C (µg/mL)
C2.5PC	CBD+2.5%P, 2.5%C (µg/mL)
C2.5LP	CBD+2.5%L, 2.5%P (µg/mL)
C2.5LM	CBD+2.5%L, 2.5%M (µg/mL)
C3.12C0.6LPM	CBD+3.125%C, 0.625%LPM (µg/mL)

**Table 9:** Composition of bioinks used for extrusion-based 3D bioprinting of cannabinoid-loaded mucoadhesive buccal films.

Component	Type/Description	Concentration/Amount
**Mucoadhesive polymer(s)**	HPMC	1–5% (*w*/*v*)
	Carbopol	1–5% (*w*/*v*)
**Plasticizer**	PEG 400	5, and 10% (*v*/*v*)
**Sweetener**	Mannitol	200 mg
**Drug + Cyclodextrin**	HP-β-CD with CBD, HP-β-CD with THC	20 mg/mL, 1:1 molar ratio
**Permeation enhancer**	D-Limonene, L-Menthol, α-Pinene, 1,8-Cineole	C: 3.125% (*w*/*v*); L, P, M: 0.625% (*w*/*v*) each

## Data Availability

The data supporting the findings of this study are included within the article. Additional data related to formulation and processing are not publicly available due to proprietary restrictions but may be available from the corresponding author upon reasonable request.
